# Digital Cell Counting Device Integrated with a Single-Cell Array

**DOI:** 10.1371/journal.pone.0089011

**Published:** 2014-02-13

**Authors:** Tatsuya Saeki, Masahito Hosokawa, Tae-kyu Lim, Manabu Harada, Tadashi Matsunaga, Tsuyoshi Tanaka

**Affiliations:** 1 Division of Biotechnology and Life Science, Institute of Engineering, Tokyo University of Agriculture and Technology, Tokyo, Japan; 2 Malcom Co., Ltd., Tokyo, Japan; Emory University/Georgia Insititute of Technology, United States of America

## Abstract

In this paper, we present a novel cell counting method accomplished using a single-cell array fabricated on an image sensor, complementary metal oxide semiconductor sensor. The single-cell array was constructed using a microcavity array, which can trap up to 7,500 single cells on microcavities periodically arranged on a plane metallic substrate via the application of a negative pressure. The proposed method for cell counting is based on shadow imaging, which uses a light diffraction pattern generated by the microcavity array and trapped cells. Under illumination, the cell-occupied microcavities are visualized as shadow patterns in an image recorded by the complementary metal oxide semiconductor sensor due to light attenuation. The cell count is determined by enumerating the uniform shadow patterns created from one-on-one relationships with single cells trapped on the microcavities in digital format. In the experiment, all cell counting processes including entrapment of non-labeled HeLa cells from suspensions on the array and image acquisition of a wide-field-of-view of 30 mm^2^ in 1/60 seconds were implemented in a single integrated device. As a result, the results from the digital cell counting had a linear relationship with those obtained from microscopic observation (r^2^ = 0.99). This platform could be used at extremely low cell concentrations, i.e., 25–15,000 cells/mL. Our proposed system provides a simple and rapid miniaturized cell counting device for routine laboratory use.

## Introduction

Today, cell counting is one of the most commonly performed routine laboratory tests in the field of cell biology. Recently, various types of desktop-sized automated cell counters including impedance-based [1], [2] and image-based counters [3], [4] have been developed and commercialized for routine laboratory use. These cell counters have been designed to reduce both operator error and the labor required for manual cell counting. In an image-based cell counter, cell concentration is calculated from several microscopic images obtained by automated microscopy. Single cells are morphologically distinguished from debris or cluster from the images and the cell concentrations are calculated from the number of single cells identified in microscopic area. The detectable cell concentration ranges from 1×10^5^ to 5×10^7^ cells/mL [3]. Because the measurable volumes of conventional cytometers are restricted to a certain amount, it is not possible to use these systems to measure samples with low cell concentrations (less than 10^3^ cells/mL). However, the ability to count small number of cells is becoming increasingly necessary to expand the utility in laboratories especially when using limited amounts of biological samples or preparing of cell standards for counting rare cells (e.g. circulating tumor cells or hematopoietic stem cells) [5].

As a platform for efficient image-based cell analysis that would be applicable to rare cell counting, our group has developed a micrometer-sized cavity array, termed a microcavity array, for the construction of a high-density single-cell array [6]–[9]. The microcavity array was designed as a micro-sized metallic filter for the arrangement of single cells in a two-dimensional array. By applying a negative pressure via the microcavities, the cell suspension immediately passes through the filter so that single cells are trapped on the geometry-controlled microcavities. Thousands of cells can be trapped in 60 seconds and arranged into a single-cell array with a density of up to 280 cells/mm^2^ [6]. In addition, this system can handle up to a milliliter level of sample by taking advantage of filtration-based cell entrapment. We have demonstrated that, using this microcavity array, it was possible to detect less than ten tumor cells from a 7.5 mL sample of blood [9]. However, the performances of single-cell array analyses are highly depended on the external microscopic equipment. In general, large-scale and expensive microscopes integrated with a computer-operated stage or microarray scanners are required to perform image-based cell analysis [10], which, up to this point, has limited the potential of single-cell array technology for simple and rapid cell counting.

Recently, miniaturized cell imaging systems based on microelectromechanical system technology have been developed as rapid, inexpensive, and portable cell counting platforms [11]–[18]. These platforms employ ultra-wide-field cell imaging using a charge-coupled device or complementary metal oxide semiconductor (CMOS) sensor plane without using objective lenses. We have also reported a novel miniaturized cell imaging system using a micro-partitioned thin-film transistor photosensor [19] and a CMOS sensor [20]. In these systems, two-dimensional imaging of single cells directly placed on a plane surface of the sensor allows large-field imaging of 30 mm^2^ and single-cell chemiluminescence detection within a second. These systems have the potential to provide simple and rapid cell counting platforms by simultaneous imaging of thousands of individual cells on a sensor surface.

In this study, we demonstrated a simple and rapid cell counting platform by integrating the microcavity array with a two-dimensional photosensor (the CMOS sensor). In the proposed system, the microcavity array serves two functions: one is to provide a microfilter for single-cell alignment in a single focal plane and the other is to provide a micro-aperture array screen for the projection of optical patterns on the sensor. The illumination light transmits through the microcavities to the CMOS sensor surface, resulting in a grid-like pattern image consisting of periodically arrayed bright spots. In this set up, the spots of trapped cells in the microcavity array are visualized as shadows in a grid-like pattern. The image acquisition conditions and subsequent image processing were optimized to validate the utility of the image-based cell counting. The cell count is determined by enumerating the uniform shadow patterns created from one-on-one relationships with single cells trapped on the microcavities in digital format. Our proposed system provides a simple and rapid miniaturized cell counting device for routine laboratory use.

## Materials and Methods

### Materials

The CMOS sensor composed of 2048×1536 pixels (pixel size: 3.2 µm) in an area of 6.55 mm×4.92 mm was used for imaging (DFK61BUC02; Imaging Source Europe GmbH; Bremen, Germany). Hoechst 33342 (Life Technologies Corporation; CA, USA) were used for fluorescent labeling of cells. Silpot 184 (Dow Corning Toray Co., Ltd.; Tokyo, Japan) was used as a prepolymer of polydimethylsiloxane (PDMS), and a peristaltic pump (MINIPULS3; Gilson, Inc.; WI, USA) was used to run the sample solution through the device.

### Cell Preparation

HeLa cells were cultured in Dulbecco’s modified Eagle’s medium (DMEM) supplemented with 10% fetal bovine serum (FBS) and 1% penicillin and streptomycin at 37°C in a humidified incubator containing 5% CO_2_. The harvested cells were washed and suspended in 500 µL of phosphate-buffered saline (PBS). The cells were fixed using 4% paraformaldehyde for 15 min at room temperature and stained with Hoechst 33342 (5 µg/mL). The number of cells in the suspension was determined as follows. Cell concentrations were first determined by manual counting using a hemocytometer (1.1×10^6^ cells/mL, average diameter: 17.6 µm). Rare cell samples were prepared by diluting cells to ideal concentrations of 5–4000 cells/5 µL by statistical sampling of a serial dilution. Then, 5 µL of a cell suspension–equal to the volume injected into the test sample (final volume: 200 µL)–was transferred onto a glass slide where cells were counted under a fluorescence microscope. Counting was repeated three times to determine the number of cells in the suspension.

### Fabrication of Digital Cell Counting Device

**Figure 1:**
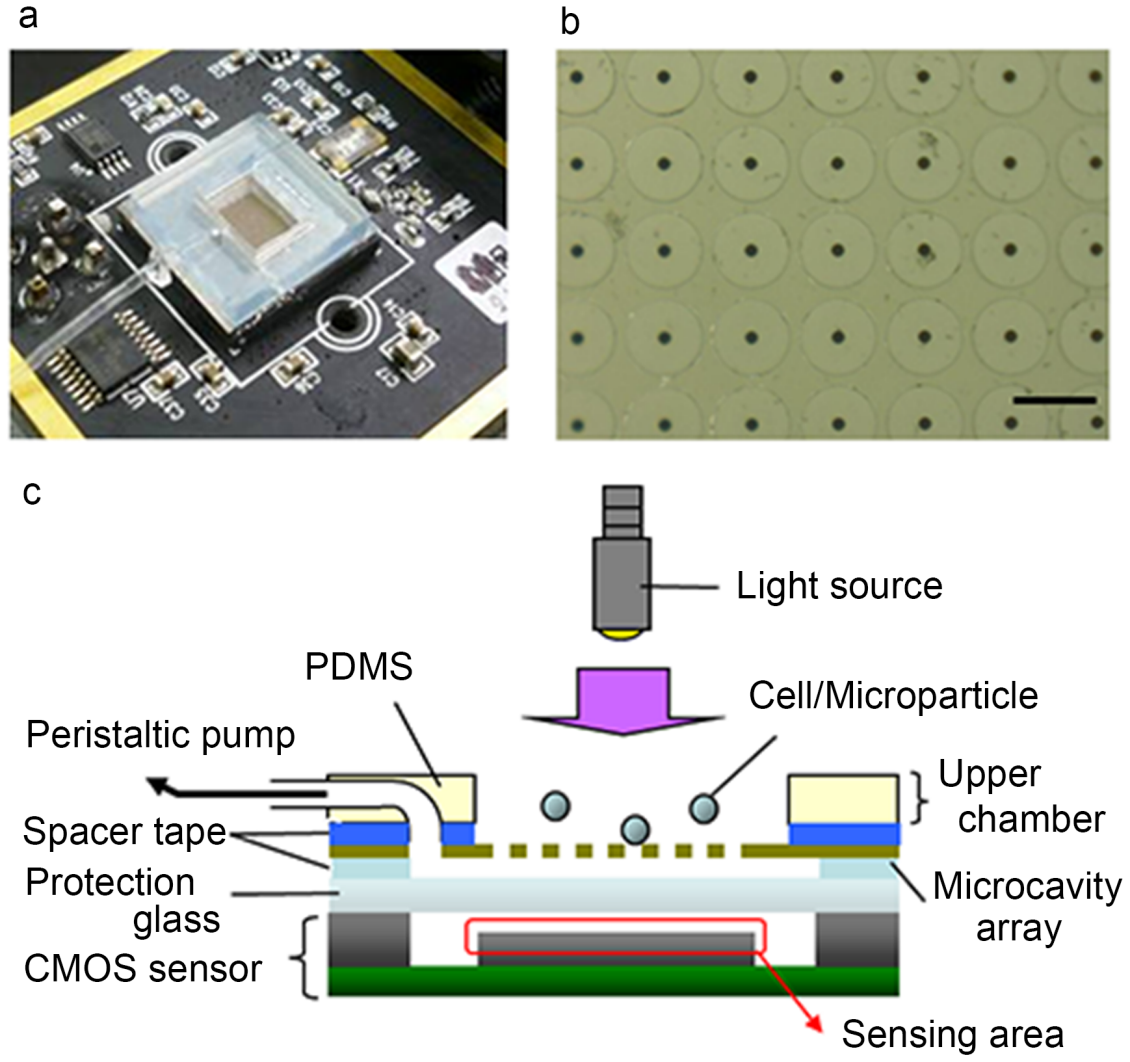
Digital cell counting device composed of a CMOS sensor and a microcavity array. (a) Photograph of the device. (b) Micrograph of the microcavity array surface. Scale bar; 60 µm. (c) Side-sectional view of the digital cell counting device.

A microcavity array was fabricated on the CMOS sensor to fabricate the digital cell counting device (Fig. 1a and 1c). The microcavity array was prepared as previously described [9]. It was made of nickel by electroforming (Optnics Precision Co., Ltd.; Tochigi, Japan). Each microcavity was fabricated with a diameter of 8 µm (Fig. 1b) and a 60-µm distance between each microcavity for achieving a total of 7,500 microcavities arranged in each 100×75 array. A suction chamber was fabricated between the back side of the microcavity array and the protection glass of the CMOS sensor using spacer tape. The chamber was connected to an outlet leading to a peristaltic pump, which was used to implement vacuum pressures on the sample fluids. An upper chamber for cell introduction (height: 1.5 mm) was fabricated on the microcavity array using PDMS.

**Figure 2:**
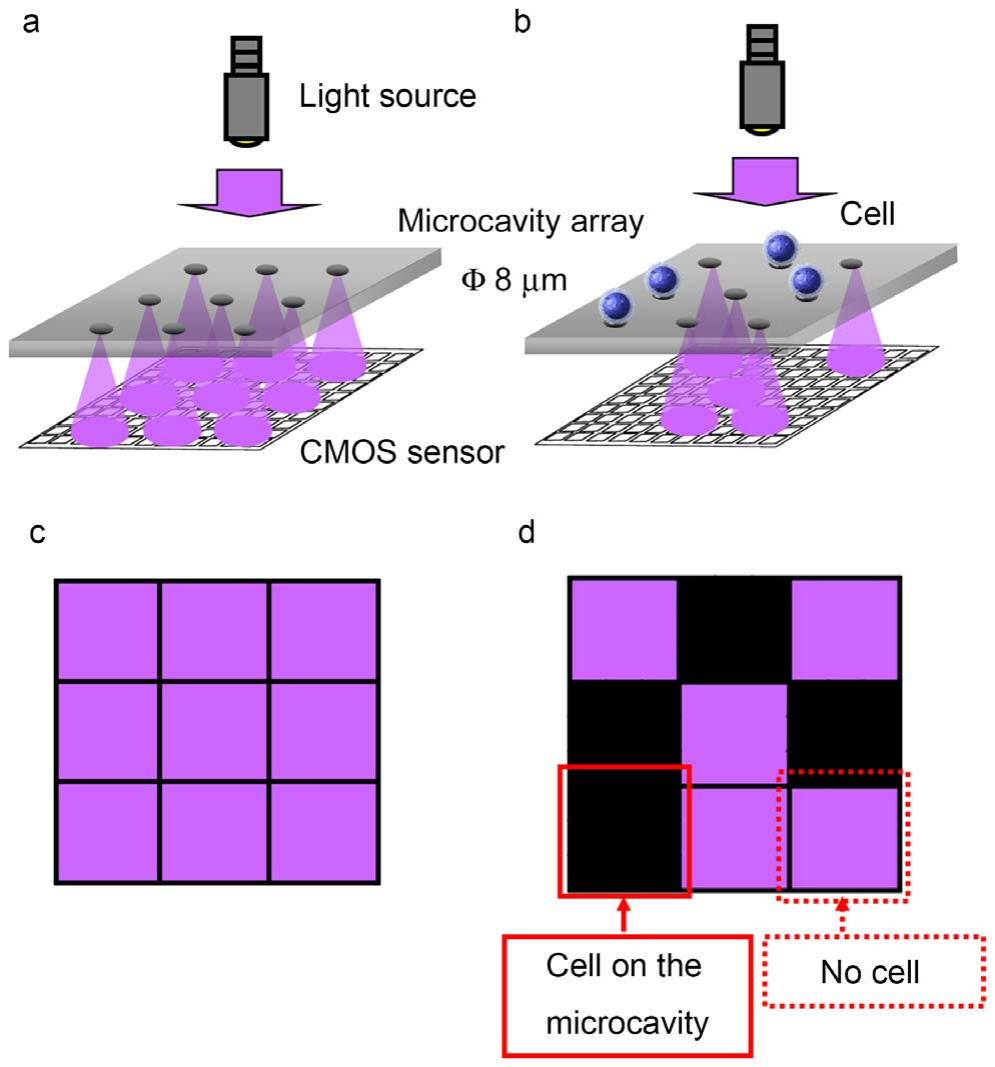
Fabrication of digital cell counting device based on a CMOS sensor integrated with a microcavity array. (a, b) Schematic diagrams of shadow-imaging platform. (a) Only the light transmitted through the microcavities positioned above the CMOS sensor is received at the sensor surface. (b) When cells are trapped on the microcavities, the light is attenuated by the trapped cells. (c, d) Schematics of the expected CMOS sensor images acquired before (c) and after cell entrapment (d).

Fig. 2 shows a schematic diagram of the shadow-imaging-based cell counting platform developed in this study. A microcavity array attached to a CMOS sensor was illuminated from above by a light source. Because the nickel substrate from which the microcavity array was constructed does not allow the transmission of light, only the light transmitted through the microcavities was received at the CMOS sensor surface (Fig. 2a and 2c). When cells were trapped on the microcavities, the light was attenuated owing to absorption and scattering (Fig. 2b). The CMOS sensor then acquired the image showing the shadow patterns derived from the light attenuation (Fig. 2d).

### Imaging of Microparticles and HeLa Cells

To evaluate the effect of separation distance from the sensor surface on imaging, fluorescent microparticles (Flowcheck Fluorosphere; Beckman Coulter, Inc.; CA, USA) with an average diameter of 10 µm were trapped and visualized by the CMOS sensor under light irradiation. The microcavity array was fabricated at various distances (i.e., 1095 µm, 1545 µm, 2045 µm) from the sensor surface. Microparticle suspensions were introduced into the well chamber. After suctioning of the suspension from below the microcavity array, the surface was illuminated with UV light (wavelength at peak emission: 365 nm, irradiance: 2 mW/cm^2^, SP-9; Ushio, Inc.; Tokyo, Japan), and the image was acquired using the CMOS sensor. The sensor was operated by Imaging software IC Capture 2.1 (Imaging Source Europe GmbH; Bremen, Germany). CMOS sensor imaging was also performed under illumination at a variety of wavelengths (i.e., 365 nm, 465 nm, 520 nm, 620 nm; irradiance: 0.1–2 mW/cm^2^). To demonstrate the use of the platform for cell counting, 200 µL of HeLa cell suspensions were introduced onto the microcavity array. After suctioning of the cell suspension at a flow rate of 120 µL/min, the microcavity array was imaged using the CMOS sensor. For determination of the captured cell count, the Hoechst-33342-stained HeLa cells were visualized using a fluorescence microscope (BX61; Olympus Corporation; Tokyo, Japan) equipped with a 10× objective lens and an automatic stage system.

## Results

### Investigation of Imaging Conditions for the Digital Cell Counting Device

**Figure 3:**
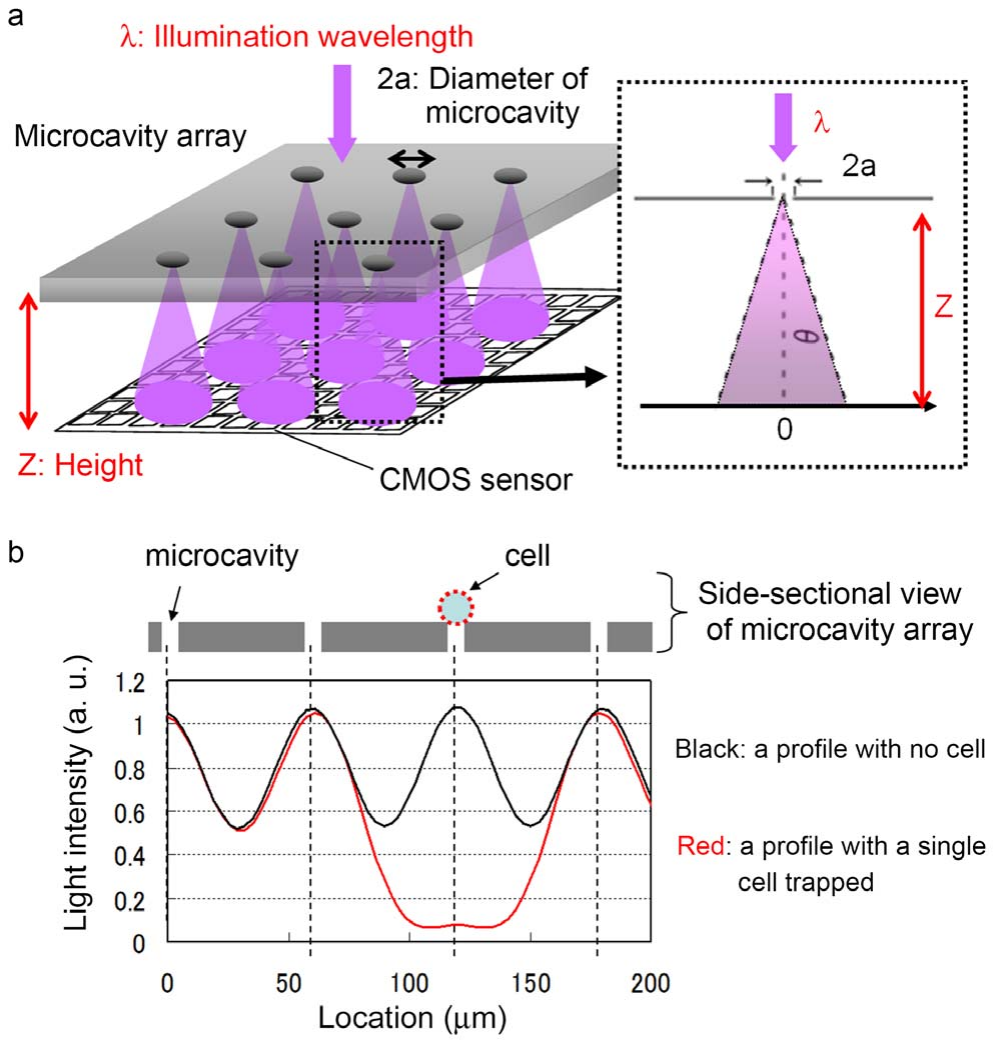
Calculated light intensity received at the sensor surface. (a) Schematic model of the shadow imaging. (b) Simulated profile of light intensity received at the sensor surface below the microcavity array. The black line indicates a profile with no cells present in the closely aligned microcavities. The red line indicates a profile with a single cell trapped on one of the closely aligned microcavities. *I_0_* was defined as 1.0. The light through the trapped cell was assumed to be attenuated by 100%.

The design of the microcavity array (diameter and pitch), illumination wavelength, and distance between the sensor and the microcavity array are critical parameters for acquisition of a high-contrast image. In this study, the microcavity array was designed for entrapment of mammalian cells with an 8 µm diameter and 60 µm pitch. The light intensities received at the sensor surface can be expressed as
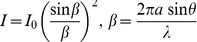
(1)where *I* is the light intensity at angle *θ* from the center of the microcavity; *I_0_* is *I* at *θ* = 0; 2*a* is the diameter of the microcavity; and λ is the illumination wavelength (Fig. 3a). Based on this equation, the cross-sectional profile of light intensity received at the sensor surface, which is 1,095 µm from the microcavity array, was simulated as 2a = 8 µm and λ = 365 nm (Fig. 3b). When no cells are on the closely aligned microcavities, light diffracted through microcavities may interfere with light through other microcavities before it is received at the sensor surface. Thus, the light intensity received by the sensor varies with the distance from the center of microcavity position, as indicated by the black line in Fig. 3b. Conversely, when a single cell is trapped on one of the closely aligned microcavities, the light is attenuated by the trapped cell. If the light through the trapped cell is diminished by 100%, the sensor element just below the trapped cell receives only the light diffracted through neighboring microcavities, as depicted by the red line in Fig. 3b. As a result, the cell entrapment position is recognized as a shadow pattern, which is darker than the surrounding image area. In order to acquire this shadow pattern image with optimized contrast, the illuminated wavelength (λ) and sensor-microcavity array distance (Z) were varied, and the differences in results were evaluated.

**Figure 4:**
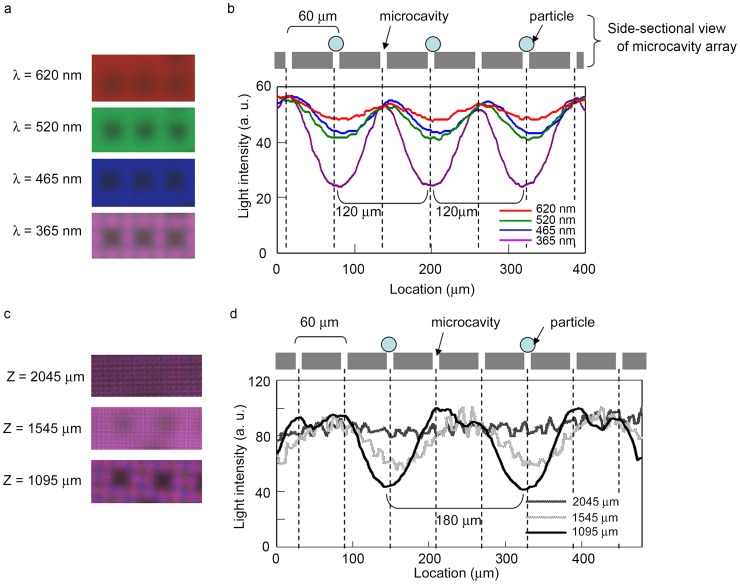
Effects of wavelength and distance between the microcavity array and sensor surface on shadow patterns. (a, c) Parts of the CMOS sensor images corresponding to selected rows of microcavities acquired under illumination at various wavelengths in constant distance: 1,095 µm (a) and at various distances in constant wavelength: 365 nm (c). Three of the seven microcavities in a row (a) and two of the eight microcavities in a row (c) were occupied by microparticles. (b, d) Cross-sectional variation in light intensity was measured in each image.

The effects of wavelength (λ) and distance (Z) on image contrast were evaluated using microparticles. Fig. 4a shows part of a CMOS sensor image of microparticles trapped on the microcavity array under illumination at various wavelengths. The distance (Z) was set at 1095 µm. Each image corresponds to a row of seven microcavities. Of the seven microcavities, the three microcavities occupied by microparticles were visualized as dark blocks. The light transmitted through the unoccupied microcavities was visualized in the color corresponding to the source wavelength. Fig. 4b shows the cross-sectional intensity profile of the CMOS image. The profile indicates that the intensity was attenuated every 120 µm, which is a distance equal to approximately two intervals. Furthermore, Fig. 4c shows CMOS sensor images acquired with the microcavity array positioned at various distances from the sensor surface at a constant wavelength of 365 nm. The distance was controlled using spacer tapes with thicknesses of 50, 500, and 1,000 µm on the protection glass, which was 1,045 µm from the sensor surface. Each image corresponds to a row of eight microcavities. Of these eight microcavities, the two microcavities occupied by microparticles were visualized as dark blocks. The cross-sectional intensity profile of the CMOS image indicates that the intensity was attenuated every 180 µm, which is approximately equal to three intervals (Fig. 4d). Of the conditions that were evaluated, *λ* = 365 nm and *Z* = 1095 µm were found to be optimal for the acquisition of cell patterns with high contrast.

### Image Processing for Cell Counting

**Figure 5:**
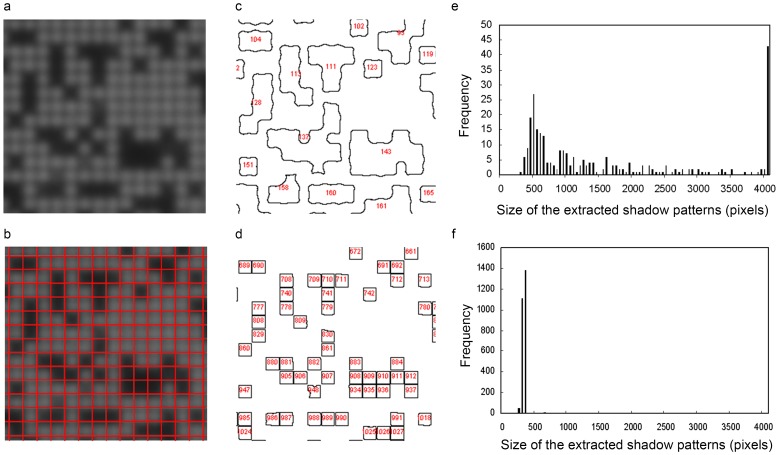
Image processing for cell counting. (a, b) The raw CMOS sensor image (a) and image merged with a pattern mask to be divided into uniform blocks (b) were processed by threshold adjustment and size extraction. (c, d) The shadow patterns were extracted from the raw image (c) and the masked image (d). Figures in red within (c, d) indicate the serial number of the extracted patterns. (e, f) Size histograms of the extracted shadow patterns were also generated.

Using the above imaging conditions, HeLa cells were trapped on the microcavity array and imaged using the CMOS sensor. The raw image was acquired using a CMOS sensor covered by a microcavity array with 7,500 microcavities arranged in a 100×75 grid at 60-µm intervals. In theory, the shadow patterns therefore appear every 60 µm within the captured image. Fig. 5a shows the shadow imaging results of the HeLa cells trapped on the microcavity array. The raw CMOS sensor images show the shadow patterns, which indicate the locations of trapped cells. For cell counting, the raw CMOS sensor image converted to 8 bit grayscale was processed by threshold adjustment and size extraction. An area measuring under 70 arbitary unit in light intensity and over 200 pixels in size was extracted. As a result of image processing, the extracted shadow patterns derived from nearby cells overlapped (Fig. 5c). In addition, the area-size histogram indicated heterogeneity in the sizes of the extracted shadow patterns (Fig. 5e). From this image, we could not achieve accurate cell counting at the single-cell resolution because of an inability to identify single shadow blocks. To address this problem, the raw CMOS sensor image was merged with a pattern mask to divide it into 100×75 uniform blocks (Fig. 5b). After masking the raw CMOS sensor image, image processing was performed by threshold adjustment and size extraction. In the processed image, each extracted shadow pattern was standardized into a square (Fig. 5d). In contrast to the raw image, the area-size histogram generated from the masked image had a narrow peak at around 350 pixels corresponding to the area size of the uniform block consisting of 19×19 pixels (Fig. 5f). These results indicate that it was possible to perform automated counting of individual cells based on shadow patterns extracted from a CMOS sensor image.

Using the same conditions (*λ* = 365 nm and *Z* = 1095 µm), microparticles with a diameter of 10 µm, JM cells (which are derived from human T lymphocytes) with diameter of 8–15 µm, and HeLa cells with diameter of 15–20 µm were trapped and their shadow patterns acquired. Microparticles (Fig. 4a), JM cells (Fig. S1b), and HeLa cells (Fig. 5a) trapped on the microcavity array cast shadow patterns with high contrast. Furthermore, the cross-sectional intensity profile of the CMOS image indicates that the intensity just below the trapped cells was attenuated (Fig. 4b, S1c). The attenuation rate, which is the ratio of the intensity just below the trapped cells to the intensity just below the microcavities without cells, was 0.45 for microparticles, 0.45 for JM cells and 0.42 for HeLa cells, whereas the rate for the simulation result was <0.1. Though the experimental attenuation rate indicates that the light is not fully attenuated by the trapped cells, these shadow patterns are independent of cell type or size and have enough contrast to be extracted and counted.

### Evaluation of Cell Counting Accuracy

**Figure 6:**
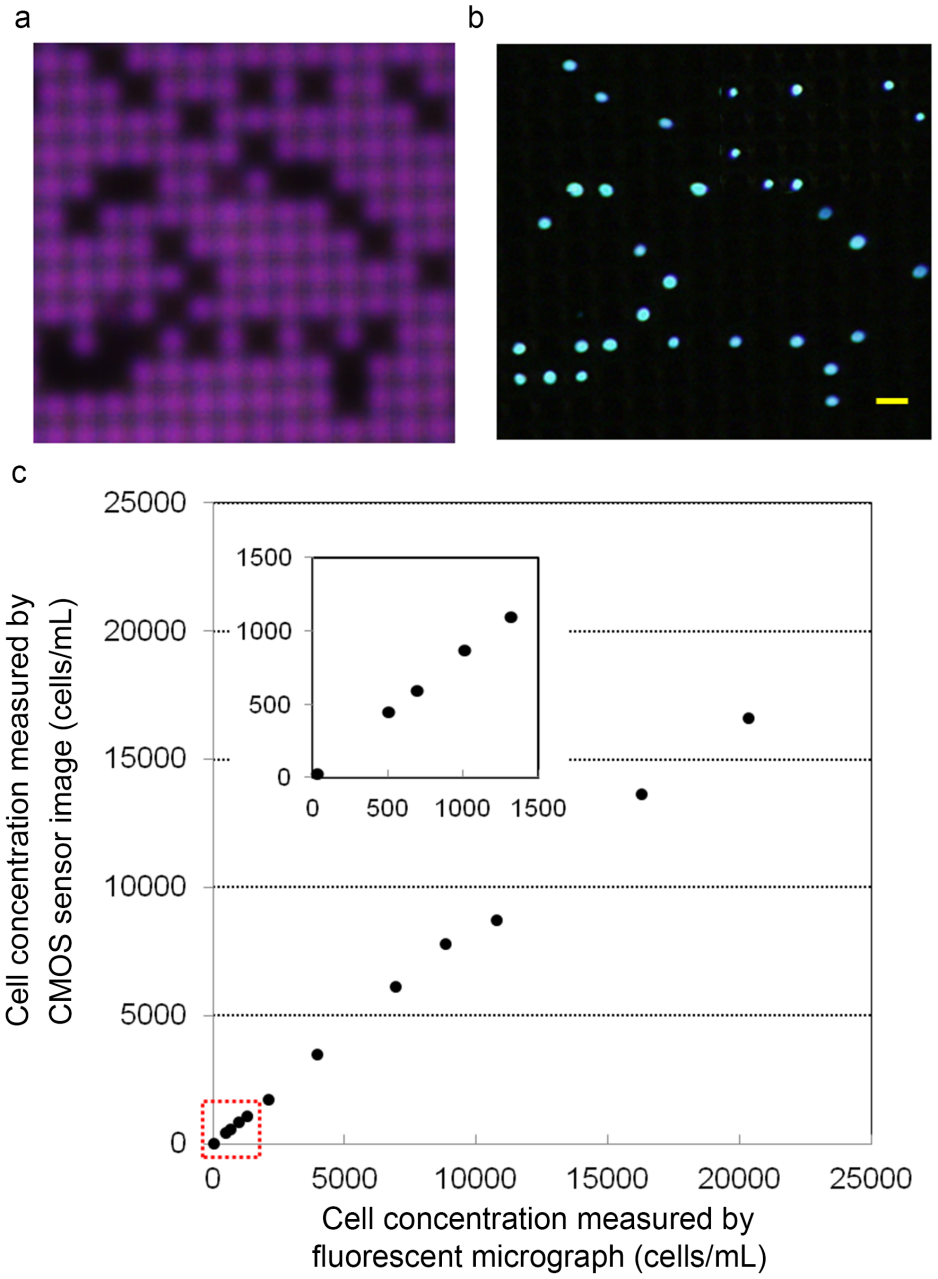
Evaluation of cell counting accuracy. CMOS sensor image (a) and fluorescent micrograph (b) of HeLa cells trapped on a microcavity array. Scale bar; 60 µm. (c) Cell count measured by CMOS sensor images compared with the count of HeLa cells stained with Hoechst 33342 in the fluorescent micrograph. Plot area in lower range (red box) is enlarged and shown on the top left side.

To demonstrate the capability of this imaging platform for cell counting, it was used to examine 200 µL HeLa cell suspensions of various cell densities. After cell entrapment was performed, the microcavity array was imaged under illumination using the CMOS sensor. In this study, cell aggregation was prevented by pipetting. In addition, the probability of entrapment of multiple cells within a single cavity was <0.1%, as was demonstrated in our previous work [7]. Almost all cells were trapped on the microcavities as single cells. Therefore, each microcavity was blocked by only one cell. In the acquired CMOS sensor images, the cell locations on the microcavity array were visualized as shadow patterns (Fig. 6a). The shadow patterns were shown to match the locations of HeLa cells stained with Hoechst 33342 and counted using the fluorescent microscope (Fig. 6b). Finally, to validate the usefulness of the cell counting platform, cell populations of different densities were trapped and measured using CMOS sensor imaging. The results showed that, over a range of 25–15,000 cells/mL, it was possible to perform, cell counting with an accuracy of 0.82, and the results obtained with this method showed a linear relationship (r^2^ = 0.99) with those obtained by microscopic observation (Fig. 6c). The discrepancy in results from the two methods mainly resulted from cells not being trapped on the microcavities. However, as the recovery rate was constant (80–90%) [21] leading to the high linearity of the relationship between the two methods, a calculation could be used to compensate for the discrepancy in the measured cell density. These results indicate that, over a range of 25–15,000 cells/mL, trapped cells could be successfully counted by the CMOS sensor based on their shadow patterns in a few minutes.

## Discussion

In our previous study, mammalian cells were directly introduced onto a two-dimensional photosensor surface, and the cell signals were detected by the light-scattering of cells [20]. Because the cell positions in the z-axis direction were not controlled on the flat surface, the light-scattering signals varied according to the distance between the cells and the sensor surface, resulting in underestimation of the cell numbers. In addition, overlapping cells were difficult to count precisely. Seo et al. (2009) also developed a lensless, ultra-wide-field cell imaging platform based on holographic diffraction [16]. In this platform, the two-dimensional holographic diffraction signature of each cell on a CMOS sensor was recorded without using objective lenses by controlling the spatial coherence of the illumination source. The recorded holographic signature was reconstructed using a custom-developed algorithm. The process permits computational separation of cell holograms in which dense cells such as red blood cells in whole blood samples are completely overlapping on the CMOS sensor [15].

In contrast, using our approach, individual cells are arranged at the single-cell level on the microcavity array so as to avoid overlapping cells, and without losing focus. As reported in our previous papers [9], the diameter of microcavities was set at 8-µm, which is suitable for trapping epithelial cell lines such as HeLa cells. The microcavity array was designed to separate and trap up to 7,500 cells above the 30 mm^2^ sensing area of the CMOS sensor. This setup enables us to detect single-cells in a simple fashion. When illuminated from above the microcavity array, the CMOS sensor acquires shadow patterns derived from light attenuation at the microcavities occupied by single cells. In order to distinguish adjacent shadow patterns at a single-cell resolution, a mask image of the grid pattern facilitated segmentation of the shadow patterns as individual squares of uniform area. The segmented shadow patterns are independent of cell size but are created from one-on-one relationships with single cells trapped on the microcavity array.

The microcavity array is made of a nickel substrate, which does not allow the transmission of light outside the microcavities. However, as D’Antò et al. reported, exposure to NiCl_2_ causes a dose- and time-dependent inhibition of cell proliferation in human cell lines [22]. Therefore, the biocompatibility of the nickel substrate with respect to cell viability or cell proliferation should be assessed in a future study, although this study was not aimed at monitoring cell proliferation but at counting trapped cells. Our previous study indicated that the use of the microcavity array does not affect HeLa cell proliferation on the surface (unpublished data), although the release of nickel ions from microcavity array substrate was not examined. These findings show that a microcavity array made with a nickel substrate does not cause immediate cell death and can therefore be used in a cell counting platform.

Another important feature of our platform is its capability for wide-field imaging of the entire 30 mm^2^ area of the microcavity array. The image used to determine the number of cells trapped on the microcavity array is acquired by the CMOS sensor in a single image capture without using objective lenses. Wide-field imaging allows counting of a small amount of cells without the need for scanning. Based on these features, the platform described in this work can be used with low cell concentrations ranging from 25 to 15,000 cells/mL, which is lower than that measurable by conventional cell counters.

However, some limitations in the proposed technique need to be addressed. When excessive numbers of cells (over 7,500 cells) were introduced into the microcavity array, the number of cells remaining on the substrate outside the microcavities increased, resulting in underestimation of the cell number. Thus, when measuring a sample with a high cell concentration, dilution is required before introducing the sample into the device. High-concentration samples could also be counted by increasing the number of microcavities and the size of the sensing area. Furthermore, use of the proposed system is limited to pure populations of cultured cell lines. The device cannot discriminate between cells and debris particles from the acquired shadow image. Hence, prevention of cell aggregation by trypsinization and pipetting are required for accurate cell counting of uniformly sized mammalian cells. Cells must then be suspended in a pre-filtered buffer because contamination by debris particles may cause false positive counting. To address these limitations, a functionalized microcavity array would be useful. For instance, in previous studies, we developed a microcavity array designed for size- and deformability-based separation of tumor cells [9] or leukocytes [7], [8] from whole blood. These studies showed that it is possible to use the microcavity array to isolate and detect specific cells on two-dimensional surfaces. Therefore, the digital cell counting device has the potential to be used for clinical applications such as leukocyte counting, although further optimization of the device design is required for it to be capable of distinguishing heterogeneous cell populations.

## Conclusions

This study presents a novel cell counting device using a two-dimensional photosensor (CMOS sensor) integrated with a microcavity array. One of the important features of the proposed technique is its capability for wide-field imaging of the entire 30 mm^2^ surface area of the single-cell array. The images are acquired by the CMOS sensor in a single image capture without the use of objective lenses or scanning. Therefore, only a few minutes were needed to complete the cell count. Furthermore, the microcavity array enables efficient entrapment of a small amount of cells from a low-density suspension. Due to these features, the proposed device can be used for counting with extremely low concentrations of cells, i.e., 2.5×10^1^ cells/mL. Our system provides a simple and rapid miniaturized cell counting device designed for routine laboratory use that has a minimum detectable cell concentration lower than that of a hemocytometer or commercially available cell counters.

## Supporting Information

Figure S1 **Cross-sectional variation in light intensity measured using JM cells.** (a) A section of the fluorescent micrograph of the microcavity array. Three of seven microcavities in a row were occupied by JM cells. (b) A section of the CMOS sensor image corresponding to the selected rows of microcavities acquired under illumination at 365 nm at 1095 µm. (d) Cross-sectional variation in light intensity was measured in the CMOS image.
(TIF)

## References

[1] Wu Y, Benson JD, Critser JK, Almasri M (2010) Note: Microelectromechanical systems Coulter counter for cell monitoring and counting. Rev Sci Instrum 81: 076103. 20687769 10.1063/1.3462327

[2] Peng L, Wang W, Bai L (2007) Performance evaluation of the Z2 coulter counter for WBC and RBC counting. Int J Lab Hematol 29: 361–368. 17824917 10.1111/j.1751-553X.2007.00868.x

[3] Huang LC, Lin W, Yagami M, Tseng D, Miyashita-Lin E, et al (2010) Validation of cell density and viability assays using Cedex automated cell counter. Biologicals 38: 393–400. 20185335 10.1016/j.biologicals.2010.01.009

[4] Berkes CA, Chan LL, Wilkinson A, Paradis B (2012) Rapid quantification of pathogenic fungi by Cellometer image-based cytometry. J Microbiol Methods 91: 468–476. 22985717 10.1016/j.mimet.2012.09.008

[5] Zhao Y, Schiro PG, Kuo JS, Ng L, Chiu DT (2009) Method for the accurate preparation of cell-spiking standards. Anal Chem 81: 1285–1290. 19115959 10.1021/ac802250d

[6] Hosokawa M, Arakaki A, Takahashi M, Mori T, Takeyama H, et al (2009) High-density microcavity array for cell detection: single-cell analysis of hematopoietic stem cells in peripheral blood mononuclear cells. Anal Chem 81: 5308–5313. 19485404 10.1021/ac900535h

[7] Hosokawa M, Asami M, Nakamura S, Yoshino T, Tsujimura N, et al (2012) Leukocyte counting from a small amount of whole blood using a size-controlled microcavity array. Biotechnol Bioeng 109: 2017–2024. 22367741 10.1002/bit.24471

[8] Hosokawa M, Asami M, Yoshino T, Tsujimura N, Takahashi M, et al (2013) Monitoring of benzene-induced hematotoxicity in mice by serial leukocyte counting using a microcavity array. Biosens Bioelectron 40: 110–114. 22770906 10.1016/j.bios.2012.06.043

[9] Hosokawa M, Hayata T, Fukuda Y, Arakaki A, Yoshino T, et al (2010) Size-selective microcavity array for rapid and efficient detection of circulating tumor cells. Anal Chem 82: 6629–6635. 20583799 10.1021/ac101222x

[10] Lindstrom S, Andersson-Svahn H (2010) Overview of single-cell analyses: microdevices and applications. Lab Chip 10: 3363–3372. 20967379 10.1039/c0lc00150c

[11] Hosseini Y, Kaler KVIS (2010) Integrated CMOS optical sensor for cell detection and analysis. Sensors and Actuators A: Physical 157: 1–8.

[12] Kang-Ho L, Jeonghun N, Sukhwan C, Hyunjung L, Sehyun S, et al (2012) A CMOS impedance cytometer for 3D flowing single-cell real-time analysis with ΔΣ error correction, Solid-State Circuits Conference Digest of Technical Papers (ISSCC), 2012 IEEE International. 19–23: 304–306.

[13] Kim SB, Bae H, Cha JM, Moon SJ, Dokmeci MR, et al (2011) A cell-based biosensor for real-time detection of cardiotoxicity using lensfree imaging. Lab Chip 11: 1801–1807. 21483937 10.1039/c1lc20098d PMC3611966

[14] Moon S, Keles HO, Ozcan A, Khademhosseini A, Haeggstrom E, et al (2009) Integrating microfluidics and lensless imaging for point-of-care testing. Biosens Bioelectron 24: 3208–3214. 19467854 10.1016/j.bios.2009.03.037 PMC2733855

[15] Seo S, Isikman SO, Sencan I, Mudanyali O, Su TW, et al (2010) High-throughput lens-free blood analysis on a chip. Anal Chem 82: 4621–4627. 20450181 10.1021/ac1007915 PMC2892055

[16] Seo S, Su TW, Tseng DK, Erlinger A, Ozcan A (2009) Lensfree holographic imaging for on-chip cytometry and diagnostics. Lab Chip 9: 777–787. 19255659 10.1039/b813943a PMC3931575

[17] Su TW, Erlinger A, Tseng D, Ozcan A (2010) Compact and Light-Weight Automated Semen Analysis Platform Using Lensfree on-Chip Microscopy. Anal Chem 82: 8307–8312. 20836503 10.1021/ac101845q PMC2987715

[18] Su TW, Seo S, Erlinger A, Ozcan A (2009) High-throughput lensfree imaging and characterization of a heterogeneous cell solution on a chip. Biotechnol Bioeng 102: 856–868. 18853435 10.1002/bit.22116 PMC4183348

[19] Tanaka T, Sunaga Y, Hatakeyama K, Matsunaga T (2010) Single-cell detection using a thin film transistor photosensor with micro-partitions. Lab Chip 10: 3348–3354. 20694269 10.1039/c0lc00039f

[20] Tanaka T, Saeki T, Sunaga Y, Matsunaga T (2010) High-content analysis of single cells directly assembled on CMOS sensor based on color imaging. Biosens Bioelectron 26: 1460–1465. 20728336 10.1016/j.bios.2010.07.081

[21] Matsunaga T, Hosokawa M, Arakaki A, Taguchi T, Mori T, et al (2008) High-efficiency single-cell entrapment and fluorescence in situ hybridization analysis using a poly(dimethylsiloxane) microfluidic device integrated with a black poly(ethylene terephthalate) micromesh. Anal Chem 80: 5139–5145. 18537270 10.1021/ac800352j

[22] D’Anto V, Valletta R, Amato M, Schweikl H, Simeone M, et al (2012) Effect of nickel chloride on cell proliferation. Open Dent J 6: 177–181. 23198004 10.2174/1874210601206010177 PMC3504722

