# Supplementary figures and images for: Digital Cell Counting Device Integrated with a Single-Cell Array
Source: PLoS One. 2014 Feb 13;9(2):e89011. doi: 10.1371/journal.pone.0089011 (PMC3923895; doi:10.1371/journal.pone.0089011)

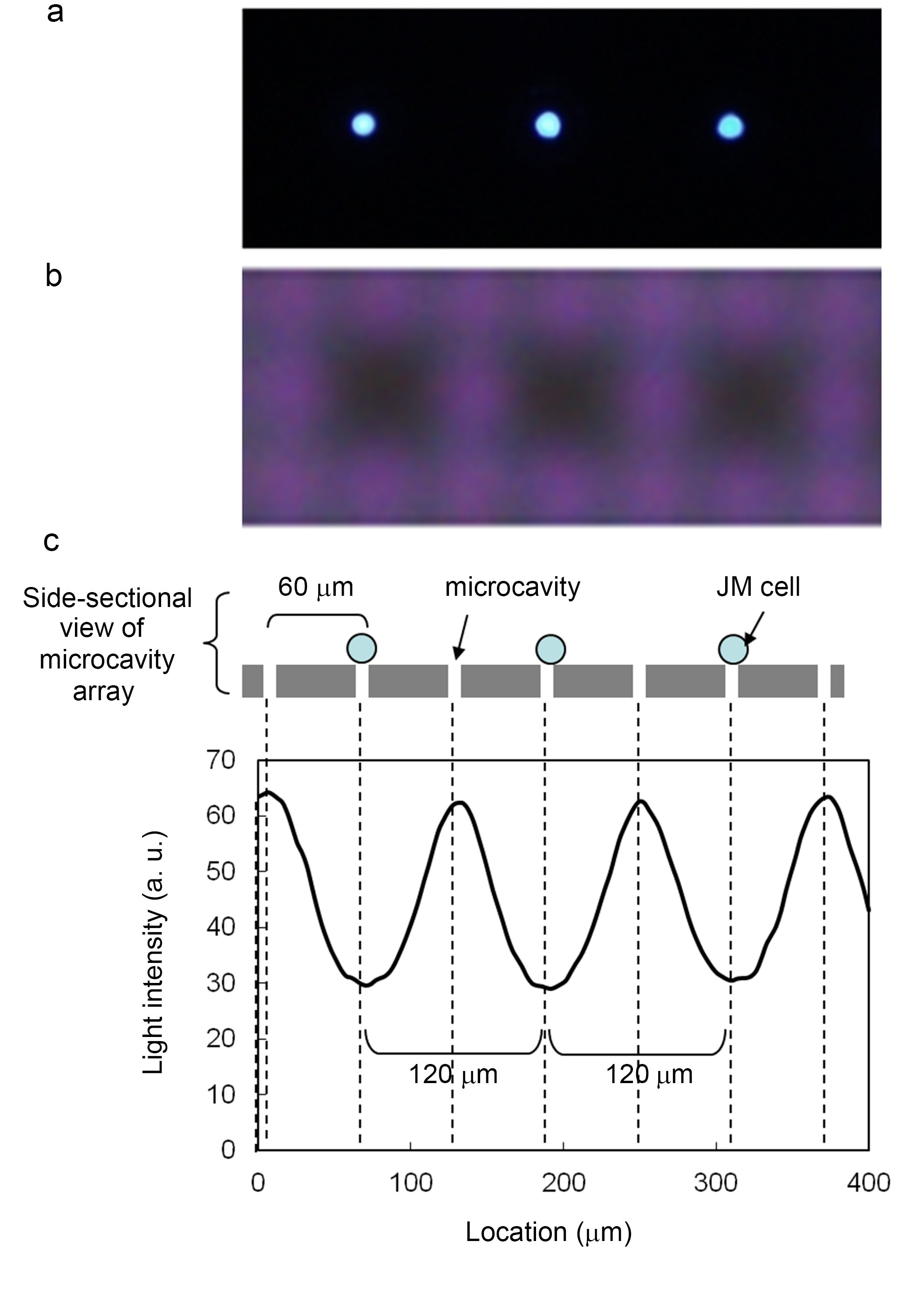

Supplement: Figure S1 — Cross-sectional variation in light intensity measured using JM cells. (a) A section of the fluorescent micrograph of the microcavity array. Three of seven microcavities in a row were occupied by JM cells. (b) A section of the CMOS sensor image corresponding to the selected rows of microcavities acquired under illumination at 365 nm at 1095 µm. (d) Cross-sectional variation in light intensity was measured in the CMOS image. (TIF) [file pone.0089011.s001.tif]
